# Characterization of the two tandem repeats for the KPC-2 core structures on a plasmid from hospital-derived *Klebsiella pneumoniae*

**DOI:** 10.1038/s41598-023-38647-z

**Published:** 2023-07-25

**Authors:** Liman Ma, Wenji Wang, Ying Qu, Dongguo Wang

**Affiliations:** 1 School of Medicine, Taizhou University, Taizhou, 318000 Zhejiang China; 2 School of Life Science, Taizhou University, Taizhou, 318000 Zhejiang China; 3 Department of Clinical Medicine Laboratory, Taizhou Municipal Hospital Affiliated with Taizhou University, 381-1 Zhongshan Eastern Road, Taizhou, 318000 Zhejiang China; 4 Department of Central Laboratory, Taizhou Municipal Hospital Affiliated with Taizhou University, Taizhou, 318000 Zhejiang China

**Keywords:** Evolution, Genetics, Microbiology

## Abstract

Today, *Klebsiella pneumoniae* strains are sophisticatedly associated with the transmission of KPC, and ST11 clones carrying KPC-2 are an important target for anti-infective clinical therapy, posing a very high threat to patients. To present the detailed genetic features of two KPC-2 core structures of F94_plasmid pA, the whole genome of *K. pneumoniae* strain F94 was sequenced by nanopore and illumina platform, and mobile genetic elements associated with antibiotic-resistance genes were analyzed with a series of bioinformatics methods. *K. pneumoniae* strain F94, identified as a class A carbapenemase-resistant *Enterobacteriaceae*, was resistant to most tested antibiotics, especially to low-levels of ceftazidime/avibactam (avibactam ≤ 4 mg/L), owing to overexpression of the two KPC-2 in F94_plasmid pA. However, strain F94 was sensitive to high-levels of ceftazidime/avibactam (avibactam ≥ 8 mg/L), which correlated with further inhibition of ceftazidime hydrolysis by the KPC-2 enzyme due to the multiplication of avibactam. Collinearity analysis indicated that multi-drug resistance (MDR) regions of plasmids with the tandam repeats of two or more KPC-2 core structures share highly similar structures. This study characterized the MDR region of the F94_ plasmid pA as homologous to plasmids pKPC2_090050, pKPC2_090374, plasmid unnamed 2, pC2414-2-KPC, pKPC2-020037, pBS1014-KPC2, pKPC-J5501, and pKPC2-020002, which contained the tandem repeats of one, two, or more KPC-2 core structures, providing insight into the evolution of multidrug resistance in *K. pneumoniae*. An alternative theoretical basis for exploring the tandem repeats of two or more KPC-2 core structures was developed by analyzing and constructing the homologous sequence of F94_ plasmid pA.

## Introduction

Carbapenem-resistant *Klebsiella pneumoniae* (CRKP) is wreaking havoc around the world and represents a significant threat to public health^1^. This threat has been emphasized by international bodies^2^, especially in North and South America, Southern and Eastern Europe, Israel, and China^3^. Clone group 258 K*. Pneumoniae* strains, including ST11, ST258, and ST512, have been implicated in KPC spread^4,5^, particularly ST11^4,6^. The KPC-containing plasmids are major genetic determinants of antimicrobial resistances^7,8^. Because of its ability to spread rapidly, the KPC-2-carrying ST11 clone is an important target of anti-infective clinical therapy and poses a very significant threat to patients^8^.

Ceftazidime/avibactam, a novel β-lactam/β-lactamase inhibitor combination with activity against KPC or OXA-48-like carbapenemases^9^, is currently one of the last antibacterial agents available to treat infections of CRKP^10^. However, *K. pneumoniae* strains with ceftazidime/avibactam resistance are rapidly emerging^11^. This is a new area challenging clinicians and researchers. Another greater and more worrying issue is that the combination of high virulence and carbapenem resistance has led to the emergence and global spread of possible “superbugs”, such as prevalent *K. pneumoniae* clone ST11^12^. Consequentially, serious and fatal community-acquired and hospital-related infections are emerging^13–15^, and these need to be actively prevented.

In accordance with the literature^6^, sequence analysis and annotation showed that plasmids carrying *bla*_KPC-2_ from clinical *K. pneumonia*e isolates harbor other antibiotic-resistance determinants, e.g., *bla*_TEM-1_, *bla*_CTX-M-65_, *bla*_CTX-M-90_, and *rmtB*, which can lead to multidrug resistance. Different mobile elements, mostly IS*26*, are located upstream and downstream of antimicrobial multi-resistance (AMR) genes and might play important roles in AMR horizontal transfer.

To date, at least 11 types of plasmid vectors encoding KPC-2 have been discovered, of which the four most populous plasmid types are IncR, IncF, IncN, and IncX^16^. Tn*3* family transposons are crucial mobile genetic elements of the KPC-2 core structures^17^. Overall, three genetic types of Tn*3* family transposons are distributed among plasmid-carried *bla*_KPC-2_ resistance determinants^18^: (i) Tn*4401*-like transposons with “*istB*-*istA*-IS*Kpn7*-*bla*_KPC-2_-IS*Kpn6*” as the core structure, such as pKPC-NY79 (GenBank accession no JX104759.1); (ii) Tn*1722*-transposons with “IS*Kpn27*-*bla*_KPC-2_-ΔIS*Kpn6-korC*” as the core structure, such as pKP048 (FJ628167.2), p628-KPC (KP987218.1), and pKPC-LK30 (KC405622.1); (iii) and those with “IS*26*-*tnpR*-IS*Kpn27*-*bla*_KPC-2_-ΔIS*Kpn6*” as the core structure, such as pECN580 (KF914891.1). In this study, we compared and analyzed MDR region of the F94_plasmid bearing KPC-2 with those of related plasmids, characterized the KPC-2 core structure of the plasmid, and explored the evolutionary mechanisms of plasmid formation.

## Materials and methods

### Bacterial strains and sequencing of the 16S rRNA gene

*K. pneumoniae* F94 strain was isolated from a sputum sample of a patient in Taizhou Municipal Hospital affiliated with Taizhou University in 2021. EC600 and *Escherichia coli* DH5α were employed as hosts for cloning. The nearly complete 16S rRNA gene of the strain was amplified by PCR using the following primers: AGAGTTTGATYMTGGCTCAG (forward) and TACCTTGTTACGACTT (Y, T, or C; M, A, or C) (reverse). The Taq enzyme was a 3:1 mixture of Fermentas Taq:Pfu (ThermoFisher Scientific, Burlington, VT, USA), and the 30 mL reaction consisted of 1.5 U of enzyme. Amplification was performed using a temperature program, including initial denaturation at 94 °C for 3 min, 30 cycles of denaturation at 94 °C for 40 s, annealing at 50 °C for 40 s, extension at 72 °C for 1 min, and final extension at 72 °C for 5 min. The length of the amplicon was about 1500 bp^19^. The PCR products were identified by bidirectional sequencing.

### Experiments of conjugal transfer and plasmid transfer

Bacterial plasmid DNA of the F94 strain was extracted using a plasmid extraction kit (TaKaRa, Dalian, China) according to the manufacturer's instructions. Plasmids were transferred from the F94 isolate into *E. coli* EC600 and DH5a by conjugation transfer and electroporation, respectively. 1000 μg/mL rifampicin and 2 μg/mL imipenem were used appropriately when selecting electroporators or transconjugates harboring the *bla*_KPC_ marker.

### Detection of Class A serine carbapenemase and Class B metallo β-lactamase

Class A carbapenemase and class B metallo β-lactamase were detected using the paper-disc agar-diffusion method recommended by CLSI^20^. The tested bacterium was prepared in a 0.5 McFarland turbidity bacterial suspension evenly spread on an Mueller–Hinton (MH) agar plate; then four imipenem discs were affixed to the surface of the agar. No liquid was added to one disc. The 3-aminophenylboronic acid (APB) solution was added to another disc at an initial concentration of 30 mg/L, gradually adding 10 μL at a time until the final concentration reached 300 μg/piece. EDTA solution was gradually added to the third piece using 10 μL of the initial concentration of 0.1 mmol/L until a final concentration of 292 μg/disc was achieved. Then APB solution (final concentration: 300 μg/disc) and EDTA solution (final concentration: 292 μg/disc) were added to all four discs at the same time. The diameters of inhibition zones were measured after overnight incubation.

The results were interpreted as follows: (1) If the diameter of the inhibition zone for the imipenem disc with APB solution was more than or equal to 5 mm more than that of the single imipenem disc, it was determined that the tested strain contained class A carbapenemase. (2) If the diameter of the inhibition zone for the imipenem disc with EDTA solution was more than 5 mm different from that of the imipenem disc alone, it was confirmed that the tested strain produced class B carbapenemase. (3) If the diameter of inhibition zone for imipenem disc onto which APB + EDTA were added simultaneously was more than 5 mm different from that of the imipenem disc alone, the tested strain carried class A carbapenemase + class B metal β-lactamase. (4) If the difference between inhibition zone diameters for imipenem discs containing enzyme inhibitor and imipenem disc alone was less than 5 mm, the tested strain did not bear class A or B carbapenemase.

### Antimicrobial susceptibility test

Bacterial resistance was detected by Bio-Merieux VITEK2 and antibiotic dilution test (MICs), and the results were determined in accordance with the 2020 CLSI Guidelines^20^. More than Twenty-one antibiotics and antibiotics + enzyme inhibitors were detected (Table 1), and *E. coli* ATCC 25922 was used as the quality control strain.

**Table 1:** Profiles of antimicrobial drug susceptibility for clinical isolate F94 of *K. pneumoniae.*

Antibiotic	MIC (mg/mL)	Genetic element	Antimicrobial type
Tobramycin	≥ 16	*rmtB*	Aminoglycoside
Amikacin	≥ 64	*rmtB*	Aminoglycoside
Ofloxacin	8	NA^a^	Quinolones
Ciprofloxacin	4	NA	Quinolones
Tigecycline	8	NA	Tetracycline
Minocycline	16	NA	Tetracycline
Cefepime	≥ 32	*bla*_KPC-2_/*bla*_CTX-M_/*bla*_TEM_	β-lactam
Ceftriaxone	≥ 64	*bla*_KPC-2_/*bla*_CTX-M_/*bla*_TEM_	β-lactam
Cefuroxime	≥ 64	*bla*_KPC-2_/*bla*_CTX-M_/*bla*_TEM_	β-lactam
Aztreonam	≥ 64	*bla*_KPC-2_/*bla*_CTX-M_/*bla*_TEM_	β-lactam
Imipenem	≥ 16	*bla*_KPC-2_	β-lactam
Ertapenem	≥ 8	*bla*_KPC-2_	β-lactam
Meropenem	≥ 16	*bla*_KPC-2_	β-lactam
Ceftazidime/Avibactam (Avibactam ≤ 4 mg/L)	≥ 16	*bla*_KPC-2_/*bla*_CTX-M_/*bla*_TEM_	β-lactam/β-lactamase inhibitor combination
Ceftazidime/Avibactam (Avibactam ≥ 8 mg/L)	≤ 2	*bla*_KPC-2_/*bla*_CTX-M_/*bla*_TEM_	β-lactam/β-lactamase inhibitor combination
Piperacillin/tazobactam	≥ 128	*bla*_KPC-2_/*bla*_CTX-M_/*bla*_TEM_	β-lactam/β-lactamase inhibitor combination
Cefoperazone/tazobactam	≥ 64	*bla*_KPC-2_/*bla*_CTX-M_/*bla*_TEM_	β-lactam/β-lactamase inhibitor combination
Amoxicillin/clavic acid	≥ 32	*bla*_KPC-2_/*bla*_CTX-M_/*bla*_TEM_	β-lactam/β-lactamase inhibitor combination
Ticacillin/clavulanic acid	≥ 128	*bla*_KPC-2_/*bla*_CTX-M_/*bla*_TEM_	β-lactam/β-lactamase inhibitor combination
Polymyxin E	≤ 0.5	NA	Polypeptide
Compound sulfamethoxazole	≤ 20	NA	Sulfonamides

^a^*NA* not applicable.

### Sequencing and sequence assembly

Genomic sequencing of strain F94 was performed on a PacBio RSII sequencer (Pacific Biosciences, CA, USA) using a sheared DNA library with an average size of 15 kb (range 10 kb to 20 kb), and on a Illumina HiSeq X sequencer (Illumina, San Diego, USA) using a paired-end library with an average insert size of 400 bp (range 150 bp to 600 bp). To improve the reliability of data processing, raw data from HiSeq X platform were trimmed to obtain the high-quality clean reads (clean data) by Canu v1.8 (https://canu.readthedocs.io/en/latest/index.html). The paired-end short Illumina reads and the long Nanopore reads were “de novo” assembled using Unicycler v0.4.5 (https://github.com/rrwick/Unicycler). Finally, accurate DNA sequences in the study were obtained. Finally, accurate DNA sequences in the study were obtained.

### Sequence annotation and comparison in detail

Open reading frames (orfs) and pseudogenes were predicted using *RAST2.0*^21^, *BLASTP/BLASTN*^22^, *UniProtKB/Swiss-Prot*^23^, and *RefSeq* databases^24^. Resistance genes, mobile elements, and other features were annotated using online databases such as *CARD*^25^, *ResFinder*^26^, IS*finder*^27^, *INTEGRALL*^28^, and the Tn Number Registry^29^. *MUSCLE 3.8.31*^30^ and BLASTN were used for multiple and pairwise sequence comparisons. The sequences of each KPC-2 core structure were compared using Mega11^31^. After removing all gaps, the evolutionary tree was constructed in RAxML^32^ and embellished with ggtree^33^, and the recombination of KPC-2 core structure sequences was detected by ClonalFrameML^34^. Visualization of genome comparisons was performed using genoPlotR^35^. Circos plot of plasmids were drawn with CGView^36^. All figures were edited using Inkscape 0.48.1 (https://inkscape.org/en).

### Nucleotide sequence accession numbers

The sequence of F94_plasmid pA was deposited on the GenBank database with an accession number ofOM144977. Comparative analysis and characteristic analysis were performed for F94_plasmid pA and related plasmids including pKPC2_090050, pKPC2_090374, Plasmid unnamed 2, pC2414-2-KPC, pKPC2-020037, pBS1014- KPC2, pKPC-J5501, and pKPC2-020002, for which the GenBank accession numbers were CP043370.1, CP066536.1, CP023942.1, CP039820.1, CP036372.1, MT269822.1, OL891656.1, and CP028541.2, respectively (Table 2).

**Table 2:** Profiles of *K. pneumoniae* plasmids examined in this study.

No	Origin of isolate	Collection date (location)	Plasmid	Status	Type	Size (kb)	GC%	GenBank accession no.
1	Patient	2016–08 (China)	pKPC2_090050	Complete	IncFII	154.724	53.08	CP043370.1
2	Environmental surface	2019–09 (China)	pKPC2_090374	Complete	IncFII	154.728	53.08	CP066536.1
3	Patient’s wound	2013–07 (Canada)	Plasmid unnamed2	Complete	IncFII	187.926	53.44	CP023942.1
4	Patient’s urine	2017–10 (China)	pC2414-2-KPC	Complete	IncFII	172.001	53.47	CP039820.1
5	Patient’s sputum	2021–01 (China)	F94 Plasmid pA	Complete	IncFII	172.743	53.57	OM144977.1
6	Patient	2017 (China)	pKPC2-020037	Complete	IncFII	172.778	53.57	CP036372.1
7	Patient’s blood	2016 (China)	pBS1014-KPC2	Complete	IncFII	170.701	53.58	MT269822.1
8	Patient	– (China)	pKPC-J5501	Complete	IncFII	105.404	53.15	OL891656.1
9	Patient	2016–08 (China)	pKPC2-020002	Complete	IncFII	177.516	53.67	CP028541.2

### Ethics approval and consent to participate

This study was approved by the Ethics Committee of Taizhou University, Zhejiang, China, and written informed consent was obtained from each of the participants in accordance with the Declaration of Helsinki. The rights of the research subjects were protected throughout, and we confirm that this study was conducted in our school. The use of human specimens and all related experimental protocols were approved by the Committee on Human Research of Taizhou University, and the protocols were carried out in accordance with approved guidelines.

## Results

### Antimicrobial susceptibility test, enzymatic property, and transferrable feature

The 16S rRNA sequence and species identification of strain F94 as *Klebsiella pneumoniae* were confirmed using BLAST and average nucleotide homology analysis of the genomic sequence. Results for the drug-susceptibility test of F94 strain were shown in Table 1. In particular, the strain also showed resistance to low-levels of ceftazidime/avibactam (avibactam ≤ 4 mg/L); nevertheless, when increasing the avibactam concentration, the strain showed sensitivity to high-levels of ceftazidime/avibactam (avibactam ≥ 8 mg/L), which was directly related to the increased avibactam further inhibiting the hydrolysis of ceftazidime by the KPC-2 enzyme.

Enzyme characterization confirmed that the strain F94, which belonged to the ST11 clone, contained only class A carbapenemase. After bacterial conjugative transfer and electroporation assays, transconjugant integrating the F94_plasmid pA could be recovered, demonstrating the successful transfer of F94_plasmid pA with class A carbapenemase, thus confirming the transferable properties of the F94_plasmid pA.

### Overview of structural features for F94_plasmid pA

The F94_plasmid pA was 172.743 kb in length and carried two replication genes *repA1* and *repA2*, and contained two multidrug resistance MDR1 and MDR2 regions, harboring resistance genes such as *bla*_CTX-M_, *bla*_TEM_, *fosA*, *rmtB* and two *bla*_KPC-2_, *merA* and four pathogenic virulence genes such as two LEE-encoded T3SS, CdpA and Rpn (pilW) (Fig. S1). Overall, there might be altered localization, insertion or deletion of multidrug-resistant gene among Tn*2*, Tn*3* and Tn*As3*. (Figs. 2, 4 and Fig. S2).

### Comparison of F94_plasmid pA with related plasmids: pKPC2_090050, pKPC2_090374, plasmid unnamed 2, pC2414-2-KPC, and pKPC2-020037, pBS1014-KPC2, pKPC-J5501, pKPC2-020002

**Figure 1:**
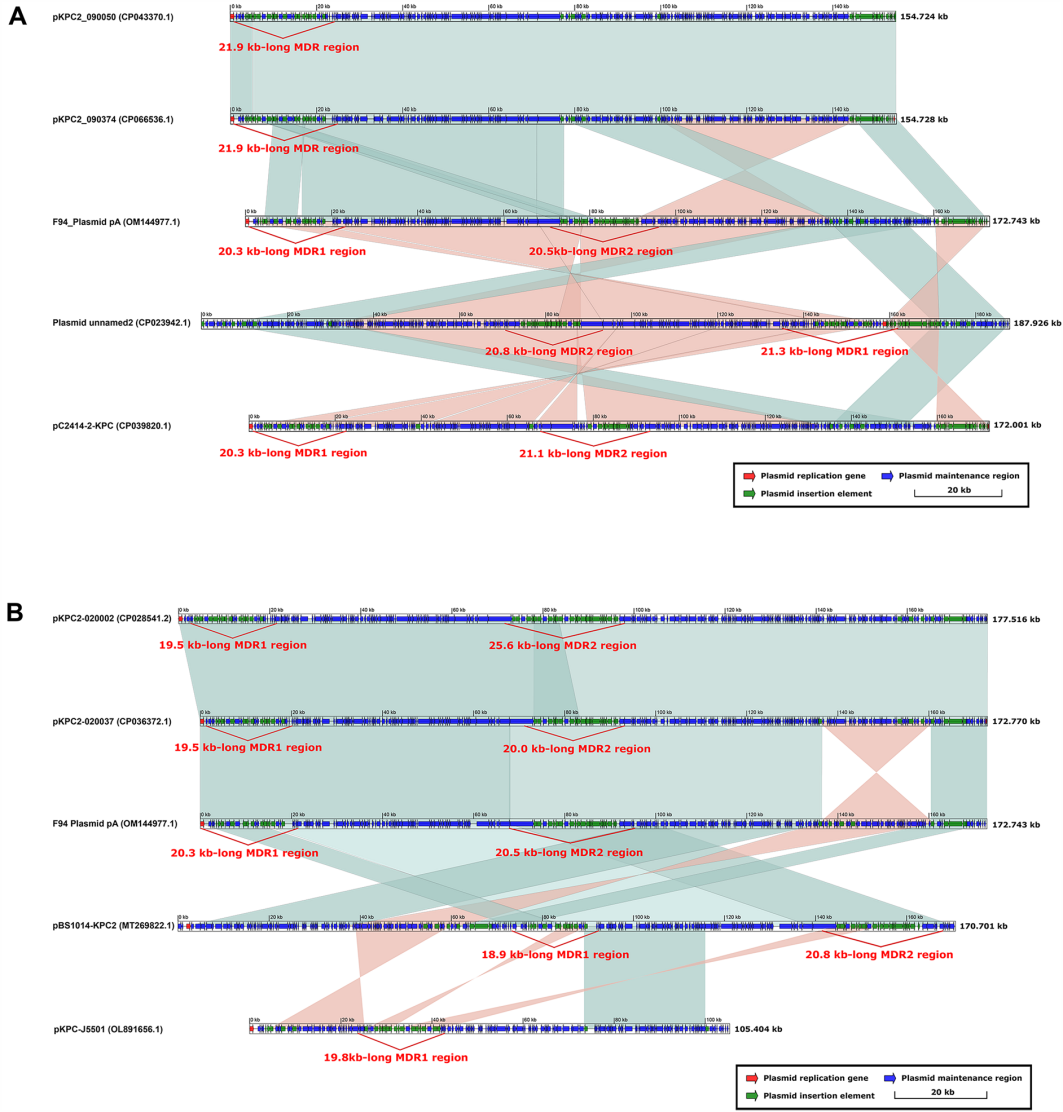
Comparison of F94_plasmid pA with related plasmids. (**A**) Comparison of F94_plasmid pA with plasmids containing single KPC-2 core structures such as pKPC2_090050, pKPC2_090374, Plasmid unnamed 2, and pC2414-2-KPC; (**B**) Comparison of F94_plasmid pA with plasmids containing the tandam repeats of two or more KPC-2 core structures, such as pKPC2-020002, pKPC2-020037, pBS1014-KPC2, and pKPC-J550. The shadow represents > 95% identity, while light blue represents the positive direction, and light pink refers to the opposite direction. Figures (**A**) and (**B**) were created by the R pacakge genoPlotR v0.8.11 software (http://genoplotr.r-forge.r-project.org/).

The sequence structures of pKPC2_090050 and pKPC2_090374 were almost identical, except that the former was 4-bp shorter, and both had one MDR region of approximately 21.9 kb in length (Fig. 1A). F94_Plasmid pA carried two MDR regions and had a regional overlapping with pKPC2_090374, which containd only one MDR region. pKPC2_090374, and F94_Plasmid pA also showed a high degree of identity, including a reverse sequence of 90–135 kb, with the corresponding region in pKPC2_090374 (Fig. 1A). Similarly, Plasmid unnamed 2 also contained two MDRs and the structure in the opposite direction and was highly consistent with F94_Plasmid pA (Fig. 1A). pC2414-2-KPC displayed a highly inverted structure and shared two MDR regions with plasmid unnamed 2 (Fig. 1A). Both pKPC2-020002 and pKPC2-020037 featured two MDRs with identical structures in the overall and overlapping regions in MDR2 (Fig. 1B). pKPC2-020037 showed excellent identity with F94_Plasmid pA in both MDRs (Fig. 1B). pKPC-J5501, with only one MDR, displayed a shorter sequence length than the above plasmids and had partial reverse identity with pBS1014-KPC2 MDR1 and MDR2 (Fig. 1B).

**Figure 2:**
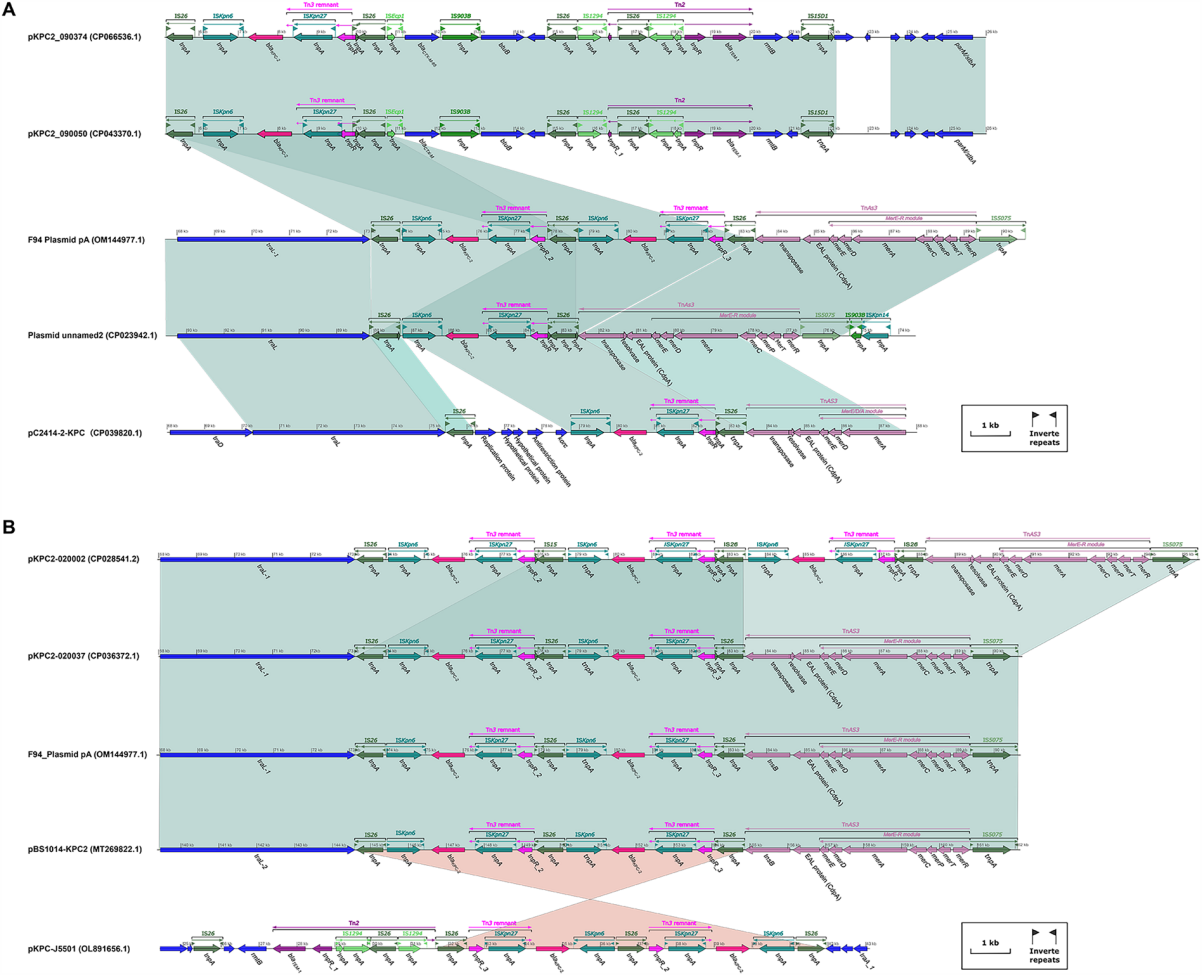
Comparison of MDR (MDR2) regions in F94_plasmid pA with those of related plasmids. (**A**) Comparison of MDR2 regions in F94_plasmid pA with those of related plasmids, such as pKPC2_090050, pKPC2_090374, Plasmid unnamed 2, and pC2414- 2-KPC; (**B**) Comparison of MDR2 regions in F94_plasmid pA with those of related plasmids, such as pKPC2-020002, pKPC2-020037, pBS1014-KPC2, and pKPC-J550. Figures (**A**) and (**B**) were also created by the R pacakge genoPlotR v0.8.11 software (http://genoplotr.r-forge.r-project.org/).

Both pKPC2_090050 and pKPC2_090374 encompassed only one MDR, including a single *bla*_KPC-2_ core structure, *bla*_CTX-M_ gene, rmt*B* gene, and Tn*2* structure. The *bla*_KPC-2_ core structure was characterized by IS*26* + IS*Kpn6* + *bla*_KPC-2_ + IS*Kpn27* + tnp*R* + IS*26*. The Tn*2* structure contained tnp*R_1* + IS*26* + IS*1294* + tnp*R* + *bla*_TEM-1_, showing that tnp*R* was divided into two parts with IS*26* + IS*1294* (two discontinuous sequences) inserted in the middle (Fig. S2A). The MDR1 of F94_Plasmid pA and pC2414-2-KPC involved a rough rmt*B* gene and Tn*2* structure, in which tnp*R* was divided into two or three parts with IS*26* to IS*1294* inserted between them (two discontinuous sequences) (approximately 10.1 kb) in F94_Plasmid pA (Fig. 2A) or IS*26* to IS*1294* (two discontinuous sequences) (approximately 6.2 kb between tnp*R_1* and tnp*R_2*) and IS*26* + hypothetical protein + *fosA* + IS*26* + IS*1294* (two discontinuous sequences) (approximately 3.7 kb between tnp*R* and tnp*R_1*) in pC2414-2-KPC (Fig. S2A). MDR1 of Plasmid unnamed 2 harbored a rough rmt*B* gene and Tn*2* structure, in which tnp*R* was divided into three parts interrupted with IS*26* to IS*1294* (5.5 kb or so) (Fig. S2A), and IS*26* + IS*1294* (approximately 0.51 kb between tnp*R* and tnp*R_1*) (Fig. S2A).pKPC2-020002 was nearly identical to pKPC2-020037 in MDR1, including a Tn*2* structure and rmt*B* gene. The Tn*2* structure was divided into three parts separated by IS*26* to IS*1294* (approximately 6.2 kb between tnp*R_2* and tnp*R_1*) and IS*26* to IS*1294* (approximately 1.3 kb between tnp*R_1* and tnp*R*) in pKPC2-020002 (Fig. S2B), or IS*26* to IS*1294* (approximately 6.2 kb between tnp*R_3* and tnp*R_2*) and IS*26* to IS*1294* (approximately 1.3 kb between tnp*R_2* and tnp*R_1*) in pKPC2-020037 (Fig. S2B). The MDR1 of pBS1014-KPC2 contained a rough rmt*B* gene and Tn*2* structure, in which tnp*R* was divided into two parts interrupted by IS*26* to IS*1294* (approximately 8.2 kb between tnp*R_3* and tnp*R_1*) (Fig. S2B). pKPC-J5501 only consisted of one MDR that was partially consistent with the reverse of MDR1 in pBS1014-KPC2. pKPC-J5501 included a rmt*B* gene and Tn*2* structure, in which tnp*R* was divided into two parts separated by IS*1294* (two discontinuous sequences) + IS*26* + IS*1294* + IS*26* between tnp*R_1* and tnp*R_3* (Tn*3* remnant) (Fig. S2B).

The MDR2 of F94_plasmid pA and other plasmids (pKPC2-020037, pBS1014-KPC2, pKPC-J5501, pKPC2-020002) comprised almost identical structures, with two or three core KPC-2 structures, where pKPC2-020002 contained three core KPC-2 structures and the others two. pKPC-J5501 harbored only one MDR, but the KPC-2 core structure was reversed compared with the others (Fig. 2B).

### Comparative analysis of all plasmids with two or more core KPC-2 structures

In addition to having exactly the same KPC-2 core structure as pKPC2-020037, pBS1014-KPC2, pKPC-J5501, and pKPC2-020002 (Fig. 2B), F94_plasmid pA also shared a similar KPC-2 core structure with pKPC2_115011, pSH9-KPC, pPA30_2, pU1121, pSRRSH1002-KPC, pYLH6_p3, pHS20R14-KPC-2, strain PA30 chromosome, and strain XHKPN39. Besides above plasmids with the tandem repeats of two or more KPC-2 core structures, some plasmids also comprised two or more KPC-2 core structures but separated by only 2–5 genes or orfs (Table 3, Fig. 5), while others possessed two or more KPC-2 core structures that were spaced apart from each other and located at different sites of the plasmids (Table 3).

**Table 3:** Profile of plasmids with two or more KPC-2 core structures.

Plasmid or chromosome (chr)	No. of core structure	Type of core structure	Source: China
The tandem repeats of two or more KPC-2 core structures			
F94 plasmid pA (OM144977.1)	2	IS*26*-(IS*Kpn6*-*bla*_KPC-2_-IS*Kpn27*-*tnpR2*-IS*26*)-(IS*Kpn6*-*bla*_KPC-2_-IS*Kpn27*-*tnpR3*-IS*26*)	Taizhou Municipal Hospital affiliated with Taizhou University
pKPC-J5501 (OL891656.1)	2	(IS*26-tnpR3-*IS*Kpn27-bla*_KPC-2_*-*IS*Kpn6*) -(IS*26-tnpR3-*IS*Kpn27-bla*_KPC-2_*-*IS*Kpn6*)-IS*26*	Yongchuan Hospital of Chongqing Medical University
pBSI014-KPC2 (MT269822.1)	2	IS*26*-(IS*Kpn6*-*bla*_KPC-2_-IS*Kpn27*-*tnpR*-IS*26*)- (IS*Kpn6*-*bla*_KPC-2_-IS*Kpn27*-*tnpR3*-IS*26*)	Sun Yat-sen University, Guangzhou
pKPC2_020037 (CP036372.1)	2	IS*26*-(IS*Kpn6-bla*_KPC-2_*-*IS*Kpn27-tnpR2-*IS*26*)- (IS*Kpn6-bla*_KPC-2_*-*IS*Kpn27-tnpR3-*IS*26*)	West China Hospital, Sichuan University, Chengdu
pKPC2_020002 (CP028541.2)	3	IS*26*-(IS*Kpn6*-*bla*_KPC-2_-IS*Kpn27*-*tnpR2*-IS*26*)- (IS*Kpn6*-*bla*_KPC-2_-IS*Kpn27*-*tnpR3*-IS*26*)- (IS*Kpn6*-*bla*_KPC-2_-IS*Kpn27*-*tnpR1*-IS*26*)	West China Hospital, Sichuan University, Chengdu
Repeats of two or more KPC-2 core structure separated by 2–5 genes or orfs			
pKPC2_115011 (CP089954.1)	2	(IS*26*-IS*Kpn6*-*bla*_KPC-2_-IS*Kpn27*-*tnpR*)-*orf1-orf2*-(IS*26*-IS*Kpn6*-*bla*_KPC-2_-IS*Kpn27*-*tnpR*)	West China Hospital, Sichuan University, Chengdu
pSH9-KPC (MH255827.1)	5	(IS*Kpn6*-*bla*_KPC-2_-IS*Kpn27*-*tnpR*-IS*26*)-*klcA-orf*-(IS*Kpn6*-*bla*_KPC-2_-IS*Kpn27*-*tnpR*-IS*26*)-*klcA-orf*-(IS*Kpn6*-*bla*_KPC-2_-IS*Kpn27*-*tnpR*-IS*26*)-*klcA-orf*-(IS*Kpn6*-*bla*_KPC-2_-IS*Kpn27*-*tnpR*-IS*26*)-*klcA-orf*- (IS*Kpn6*-*bla*_KPC-2_-IS*Kpn27*-*tnpR*-IS*26*)	Hongkong Polytechnic University, Hongkong
pPA30_2 (CP104872.1)	2	(IS*Kpn6-bla*_KPC-2_*-*IS*Kpn27-tnpR*) -*orf1-orf2-orf3-orf4*-(IS*Kpn6-bla*_KPC-2_*-*IS*Kpn27-tnpR*)	Sir Run Run Shaw Hospital, Hangzhou
pU1121 (ON614169.1)	2	(IS*Kpn6-bla*_KPC-2_*-*IS*Kpn27-tnpR*) -*orf1-orf2-orf3-orf4*-(IS*Kpn6-bla*_KPC-2_*-*IS*Kpn27-tnpR*)	The first affiliated Hospital of Zhejiang University, Hangzhou
pSRRSH1002-KPC (CP064398.1)	2	(IS*Kpn6-bla*_KPC-2_*-*IS*Kpn27-tnpR*) -*orf1-orf2-orf3-orf4*-(IS*Kpn6-bla*_KPC-2_*-*IS*Kpn27-tnpR*)	Sir Run Run Shaw Hospital, Hangzhou
Plasmid YLH6_p3 (MK882885.1)	2	(IS*Kpn6-bla*_KPC-2_-IS*Kpn27-tnpR*) -*orf1-orf2-orf3-orf4*-(IS*Kpn6-bla*_KPC-2_-IS*Kpn27-tnpR*)	Sir Run Run Shaw Hospital, Hangzhou
pHS20R14-KPC-2 (CP064771.1)	3	(*tnpR-*IS*Kpn27-bla*_KPC-2_*-*IS*Kpn6*)-*orf1-klcA-orf2-orf3-orf4*-(*tnpR-*IS*Kpn27-bla*_KPC-2_-IS*Kpn6*)-*orf1-klcA-orf2-orf3-orf4*- (*tnpR-*IS*Kpn27-bla*_KPC-2_-IS*Kpn6*)	Huashan Hospital, Shanghai
Strain PA30 chr (CP102441.1)	2	(IS*Kpn6-bla*_KPC-2_-IS*Kpn27-tnpR*) -*orf1-orf2-orf3-orf4*-(*tnpR-*IS*Kpn27-bla*_KPC-2_-IS*Kpn6*)	Sir Run Run Shaw Hospital, Hangzhou
Strain XHKPN391 chr (P066915.1)	2	(IS*Kpn6-bla*_KPC-2_-IS*Kpn27-tnpR-*IS*26*) -*orf1-klcA-orf2*-(IS*Kpn6-bla*_KPC-2_-IS*Kpn27-tnpR-*IS*26*)	Xinhua Hospital, Shanghai
Two or more KPC-2 core structures spaced apart from each other			
pXHKP309-1 (CP066901.1)	2	(IS*Kpn6-bla*_KPC-2_-IS*Kpn27-tnpR-*IS*26*) // (IS*26-*IS*Kpn6-bla*_KPC-2_-IS*Kpn27-tnpR-*IS*26*)	Xinhua Hospital, Shanghai
pKPC-063001 (MZ156798.1)	2	(IS*Kpn6-bla*_KPC-2_-IS*Kpn27-tnpR-*IS*26*) // (IS*26-ISKpn6-bla*_KPC-2_-IS*Kpn27-tnpR-*IS*26*)	Yongchuan Hospital of Chongqing Medical University
pE0171_KPC (MK370988.1)	2	(IS*26-tnpR-*IS*Kpn27-bla*_KPC-2_-IS*Kpn6*) // (IS*26-tnpR-*IS*Kpn27-bla*_KPC-2_-IS*Kpn6*)	Chinese Academy of Sciences, Beijing
pA1836-KPC (MT810353.1)	3	(IS*26-tnpR-*IS*Kpn27-bla*_KPC-2_-IS*Kpn6*) // (*tnpR-*IS*Kpn27-bla*_KPC-2_-IS*Kpn6*) //(IS*26-tnpR-*IS*Kpn27-bla*_KPC-2_-IS*Kpn6*)	Institute of Microbiology and Epidemiology, Beijing
pE02162_KPC (MK370991.1)	3	(IS*Kpn6-bla*_KPC-2_-IS*Kpn27-tnpR-*IS*26*) //(IS*Kpn6-bla*_KPC-2_-IS*Kpn27-tnpR-*IS*26*)//(IS*Kpn6-bla*_KPC-2_-IS*Kpn27-tnpR-*IS*26*)	Chinese Academy of Sciences, Beijing
Plasmid unnamed4 (CP097674.1)	2	(IS*Kpn6-bla*_KPC-2_-IS*Kpn27-tnpR-*IS*26*) //(IS*Kpn6-bla*_KPC-2_-IS*Kpn27-tnpR*)	PLA General Hospital, Beijing
pJNKPN52_KPC_FOS (MZ512197.1)	2	(*bla*_KPC-2_-IS*Kpn27-tnpR-*IS*26*) //(IS*26-tnpR-*IS*Kpn27-bla*_KPC-2_-IS*Kpn6*)	Shandong Provincial Hospital, Jinan
Plasmid unnamed2 (MZ475694.1)	2	(IS*26-tnpR-*IS*Kpn27-bla*_KPC-2_-IS*Kpn6*) //(*tnpR-bla*_KPC-2_-IS*Kpn6*)	Huashan Hospital, Shanghai
pSH2-85K-MDR (MH643792.1)	2	(IS*26-tnpR-*IS*Kpn27-bla*_KPC-2_) //(IS*Kpn6-bla*_*KPC-*_*2-*IS*Kpn27-tnpR-*IS*26*)	Hongkong Polytechnic University, Hongkong
pBSI039-KPC2 (MT269828.1)	2	*orf1*-*bla*_KPC-2_-*orf2*-(IS*26-tnpR-*IS*Kpn27-bla*_*KPC-2*_*-*IS*Kpn6*) //(*bla*_KPC-2_-IS*Kpn27-tnpR-*IS*26*)	Sun Yat-sen University, Guangzhou

**Figure 3:**
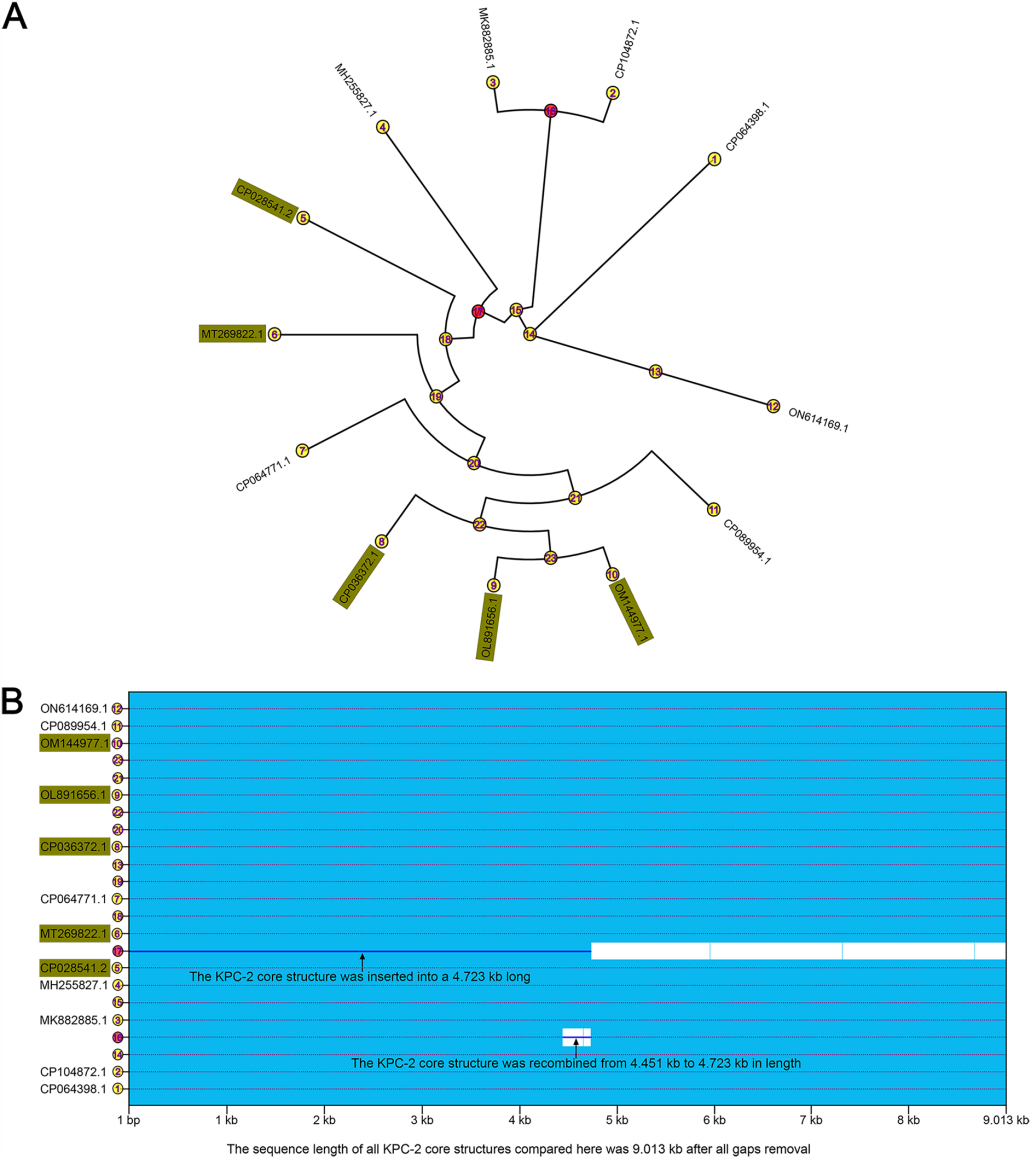
Comparison of evolutionary characteristics of the tandam repeats of two or more KPC-2 core structures and repeats of two or more KPC-2 core structures separated by 2–5 genes or orfs. 1–12 in the yellow circles indicate the sequence of KPC-2 core structures; GenBank numbers in the dark yellow boxes highlight two or more KPC-2 core structures directly in tandem; 13–23 denote nodes of the phylogenetic tree; Yellow numbers show that no recombination events have occurred, and red numbers imply that insertion or recombination events have occurred in the sequence of the KPC-2 core structure. (**A**) Diagram of phylogenetic tree for above two or more KPC-2 core structures; Figure (**A**) was constructed by RAxML v8.2.12 for the tree (https://github.com/stamatak/ standard-RAxML). (**B**) Diagram of sequence recombination analysis for above two or more KPC-2 core structures; (**B**) was build by ClonalFrameML v1.12 (https://github.com/xavierdidelot/clonalframeml).

**Figure 4:**
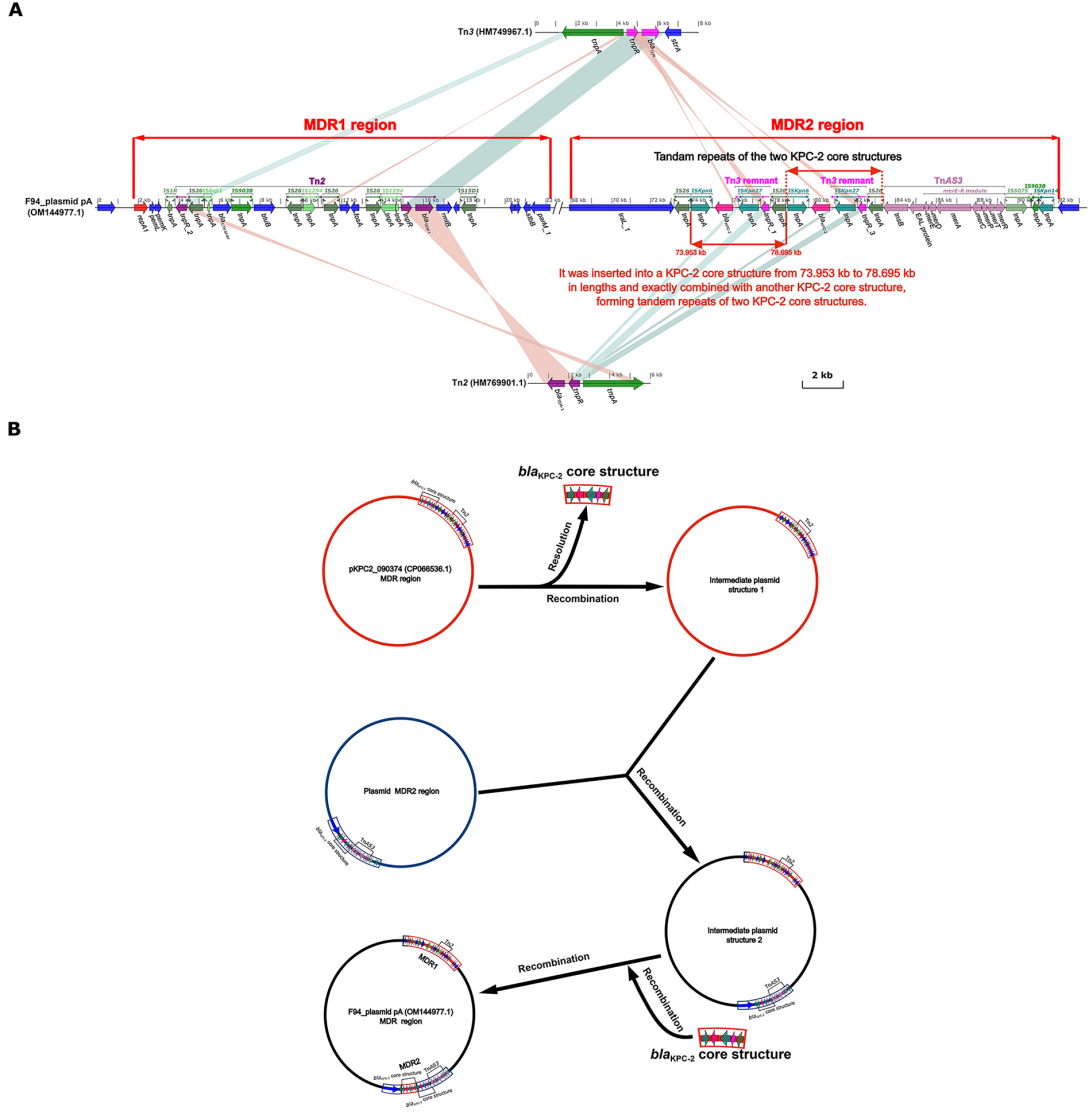
Diagram of evolutionary mechanism for F94_plasmid pA. (**A**) After comparative analysis of F94_plasmid pA with Tn*2* and Tn*3*, the basis of its MDR region fine-scale annotation was determined, and the location and rationale for forming the direct tandem of two KPC-2 core structures were identified. Figure (**A**) was created by the R pacakge genoPlotR v0.8.11 software (http://genoplotr.r-forge.r-project.org/). (**B**) Combining the above data, a reasonable hypothesis was made as to how the tandem repeats of two KPC-2 core structures of F94_plasmid pA were developped. Figure (**B**) was drawn manually using Inkscape 0.48.1 (https://inkscape.org/en).

The KPC-2 core structures of these plasmids, including F94_plasmid pA, pKPC2-020037, pBS1014-KPC2, pKPC-J5501, pKPC2-20002, pKPC2_115011, pSH9-KPC, pPA30_2, pU1121, pSRRSH1002-KPC, pYLH6_p3, and pHS20R14- KPC-2, were determined to be intrinsically evolutionarily related through a study of molecular evolutionary genetics and comparative visual analysis (Fig. 3A). The KPC-2 core structural sequences 1 to 12 shown in Fig. 3A belonged to the above mentioned plasmid sequences, while sequences 13 to 23 represented phylogenic nodes. Through a further investigation of the KPC-2 core structure of the above plasmids, we found that the inserted phylogenetic node 17 was a 4.723 kb located from 1 bp to 4.723 kb; accordingly, phylogenetic node 17 was inserted into the KPC-2 core structure located from 771 bp to 5.513 kb (Fig. S3). It can be deduced from Fig. 3A that the insertion of the KPC-2 core structure of phylogenic node 17 directly led to the formation of two or more KPC-2 core structures for real plasmids 4, 5–10 and phylogenic nodes 18 and 19–23 (Fig. 3A). It was determined that a KPC-2 core structure sequence had been inserted in F94_plasmid pA at 73.953 kb to 78.695 kb (Fig. S3), which happened to be directly connected to another KPC-2 core structure to form the tandem repeats of two KPC-2 core structures (10.197-kb length) (Figs. 4A, S3). Additionally, we found that the KPC-2 core structure of phylogenic node 16 had undergone a recombination event at 4.451 kb to 4.723 kb (Fig. 3B), which had directly led to changes before the formation of real plasmids 2 (CP107842.1) and 3 (MK882885.1) but caused no changes in F94_plasmid pA.

### Mechanism of F94_plasmid pA formation

By comparing F94_plasmid pA with Tn*2* and Tn*3*, we discovered that MDR1 in F94_ plasmid pA was formed by inserting a different insert sequence (IS) or gene with Tn*2* as a backbone, and MDR2 was developed from Tn*3* residues and Tn*As3* and included a direct tandem repeats of the two KPC-2 core structures as a backbone. F94_plasmid pA was inserted at 73,953 kb to 78,695 kb and combined with another complete KPC-2 core structure, forming the tandem repeats of two KPC-2 core structures (Fig. 4A).

## Discussion

KPC-producing *Klebsiella pneumoniae* is widely disseminated worldwide, and ceftazidime/avibactam is one of the few drugs that can effectively treat infections of these bacteria^37^. However, reports of resistance to different concentrations of ceftazidime/avibactam have progressively increased^18,38–40^. A susceptibility test for strain F94 in this study ascertained it also had resistance to ceftazidime/avibactam (avibactam ≤ 4 mg/L), but sensitivity to hight-levels of ceftazidime/avibactam (avibactam ≥ 8 mg/L) (Table 1) . One reason might be related to the tandem repeats of two KPC-2 core structures in F94_plasmid pA, which increased the expression of KPC carbapenemases. In addition, hight-levels of avibactam (avibactam ≥ 8 mg/L) further reduced the hydrolytic efficacy of KPC-2 enzyme on ceftazidime, thus making ceftazidime/avibactam exhibit a sensitive effect on F94 strain^41^. Also, the amino acid at site 179 of the two *bla*_KPC-2_ remained D (Asp) and was not substituted, therefore, we hypothesized that the strain should still manifest sensitivity to specific concentrations of ceftazidime/avibactam^41,42^, which was consistent with our results for high-levels of ceftazidime/avibactam (avibacta ≥ 8 mg/L). In general, three main causes of ceftazidime/avibactam resistance in CRKP strains have been reported worldwide, comprising the co-production of metallo-β-lactamases, the enhanced expression of KPC carbapenemases, and amino acid substitutions/deletions/ insertions in key sites of KPC carbapenemases^39,41,43^.

**Figure 5:**
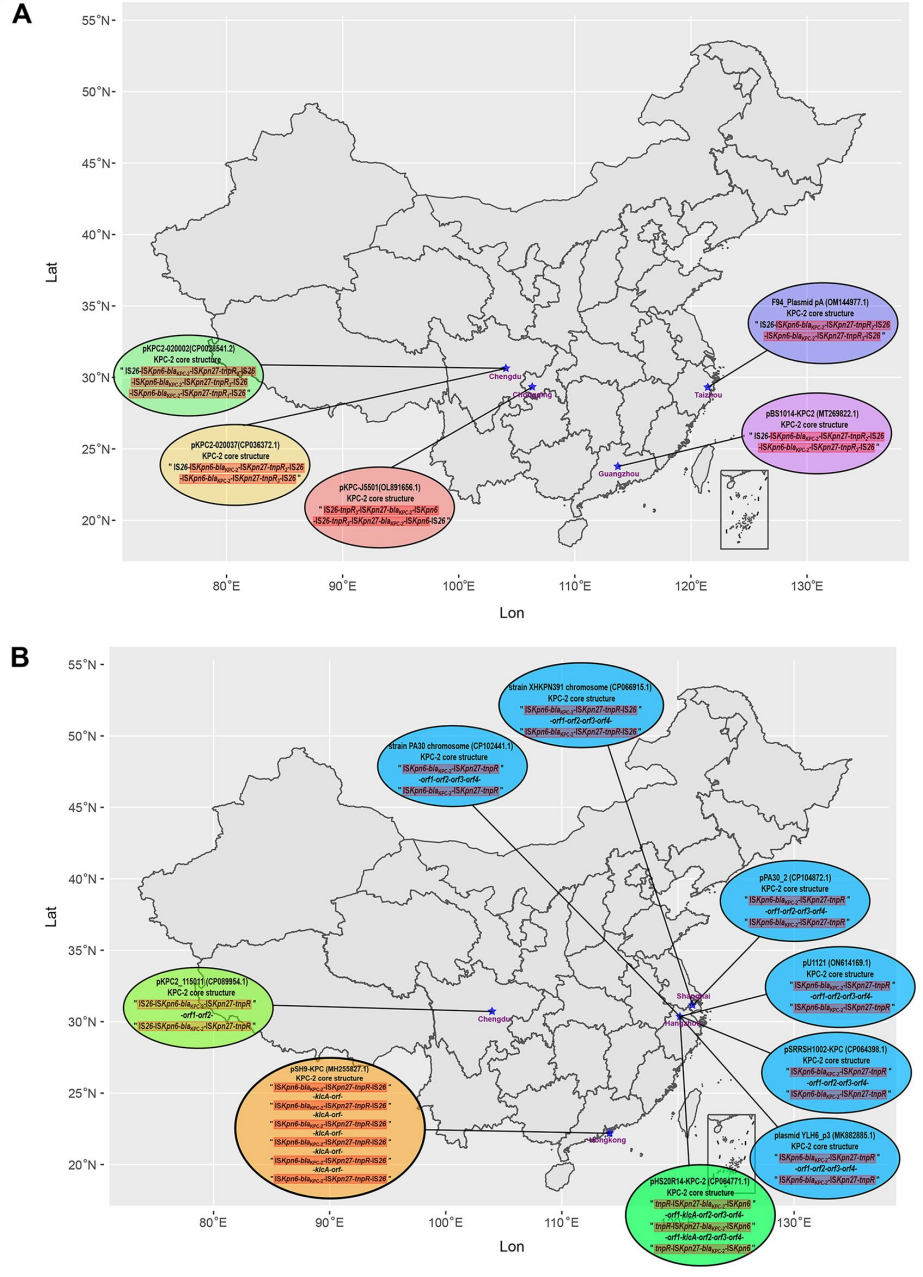
Fine localization maps and structural features of the tandem repeats of two or more KPC-2 core structures and repeats of KPC-2 core structures separated by 2–5 genes or orfs based on Table 3. (**A**) Map and structural features of the tandem repeats of two or more KPC-2 core structures; (**B**) Map and structural features of repeats of two or more KPC-2 core structures separated by 2–5 genes or orfs. The data for the map of China drawn by R package sf v1.0–9 (https://github.com/r-spatial/sf/) were obtained from http://xzqh.mca.gov.cn/map. The contents of KPC-2 core structures inside the ellipse were crafted manually using Inkscape 0.48.1 (https://inkscape.org/en).

To accurately investigate the emergence of two or more KPC-2 core structures in the study, we compared all plasmids on GenBank containing two or more KPC-2 core structures based on the tandem repeats of two KPC-2 core structures of the F94_ plasmid pA listed in Table 3, and established the intrinsic relationship model described in Fig. 3 by interrogation analysis. When a plasmid carrying a single KPC-2 core structure, such as pKPC2_090374, lost its KPC-2 core structure, it evolved into an intermediate plasmid structure 1 containing a Tn*2* structure. If the intermediate plasmid structure 1 also combined with another plasmid carrying a KPC-2 core structure, it became an intermediate plasmid structure 2 containing both a Tn*2* structure and a KPC-2 core structure. Under suitable conditions, the lost KPC-2 core structure was integrated into the site of the KPC-2 core structure (Fig. 4A) of intermediate plasmid structure 2, thus, the tandem repeats of two KPC-2 core structures, as in F94_plasmid pA, was realized (Fig. 4B). Figure 5 illustrated the features and geographic locations for two or more KPC-2 core structures that were of the tandam repeats or repeats of two or more KPC-2 core structure separated by 2–5 genes or orfs, with which the prevalence could be better characterized.

Although the plasmid recombination of multicopy *bla*_KPC-2_ had been reported^44^, however, it illustrated several KPC-2 core structures similar to the tandem repeats formed by “IS*Kpn27(8)*-*bla*_KPC-2_-ΔIS*Kpn6*-*korC*”^18^, while we inferred that the tandem repeats formed by “IS*26*-t*npR*-IS*Kpn27*-*bla*_KPC-2_-ΔIS*Kpn6*”^18^, unlike the above^18,44^. After collating all analysis results, we concluded that (1) the formation of F94_plasmid pA was the result of the long-term evolution of Tn*2*, Tn*3*, Tn*As3*, and IS through the insertion, deletion, and colonization of drug-resistance genes (Figs. 2, 4, S2); (2) Tracing their evolution revealed that the tandam repeats of two KPC-2 core structures of F94_ plasmid pA were inserted and recombined from the phylogenic node 17 into a KPC-2 core structure at 1 bp to 4.723 kb, and through a series of evolutionary steps, a daughter F94_ plasmid pA was established in which the KPC-2 core structure was inserted into another KPC-2 core structure at 73.953 kb to 78.695 kb at some time (Figs. 3, 4, S3). (3) The plasmids with two or more KPC-2 core structures in tandem repeats had intrinsic and precise connections with other plasmids involving two or more KPC-2 core structures separated by 2–5 genes or orfs (Table 3, Fig. 5), which is illustrated in Fig. 3.

## Conclusions

This study characterized the tandem repeats of two KPC-2 core structures in F94_plasmid pA and demonstrated that pKPC2_090050, pKPC2_090374, plasmid unnamed 2, pC2414-2-KPC, F94_plasmid pA, pKPC2-020037, pBS1014-KPC2, pKPC-J5501, and pKPC2-020002 had homologous sequence structures in their MDR regions, providing a unique and reliable theoretical basis for exploring the formation mechanism of the tandem repeats of two or more KPC-2 core structures. It is reasonable to assume that novel plasmids will be discovered carrying 4, 6 to 10, or even more the tandem repeats of KPC-2 core structure, or harboring 4, 6 to 10, or even more repeats of the KPC-2 core structure separated by 2–5 genes or orfs, which will pose an even greater challenge to human multidrug-resistance therapy.

## Supplementary Information

Supplementary Information 1.

Supplementary Figures.

## Data Availability

The datasets of F94_plasmidA generated during the current study are available in the GenBank repository as OM144977.
