# Supplementary material for: Characterization of the two tandem repeats for the KPC-2 core structures on a plasmid from hospital-derived Klebsiella pneumoniae
Source: Sci Rep. 2023 Jul 25;13:12049. doi: 10.1038/s41598-023-38647-z (PMC10368644; doi:10.1038/s41598-023-38647-z)
Supplement: Supplementary file 2 — Supplementary Figures. [file 41598_2023_38647_MOESM2_ESM.pdf]

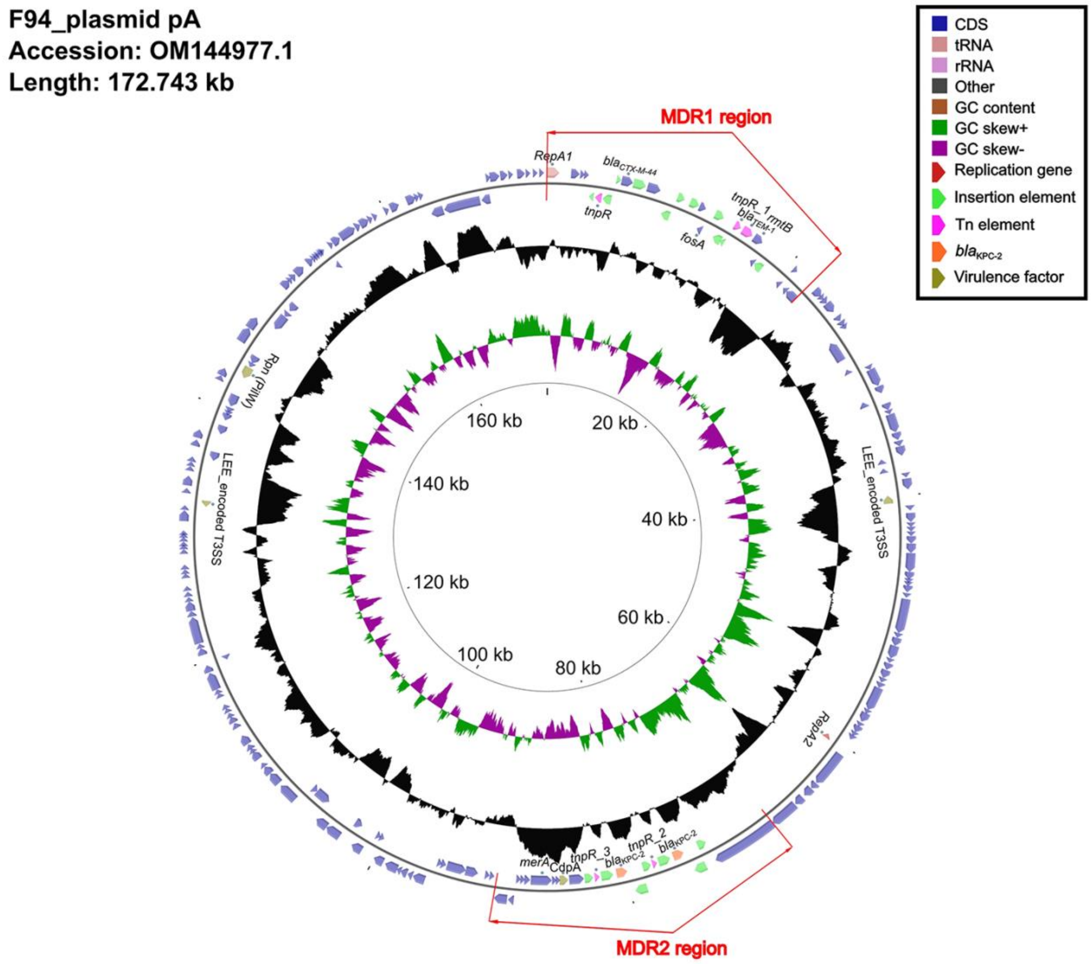

Fig. S1 Circos plot of F94_plasmid pA. Two types of MDR, including MDR1 and MDR2, were identified. MDR1 harbors drug resistance genes such as *bla*$_{\text{CTX-M-44}}$, *bla*$_{\text{TEM-1}}$, *rmtB*, and *fosA*. MDR2 contains the direct tandem of two KPC-2 core structures, such as IS*26*-(ISKpn*6*-*bla*$_{\text{KPC-2}}$-ISKpn*27*-*tnpR2*-IS*26*) - (IS*Kpn6*-*bla*$_{\text{KPC-2}}$-IS*Kpn27*-*tnpR*3-IS*26*). This diagram was established with CGview v2.0.3 (https:// github.com/paulstothard/cgview).

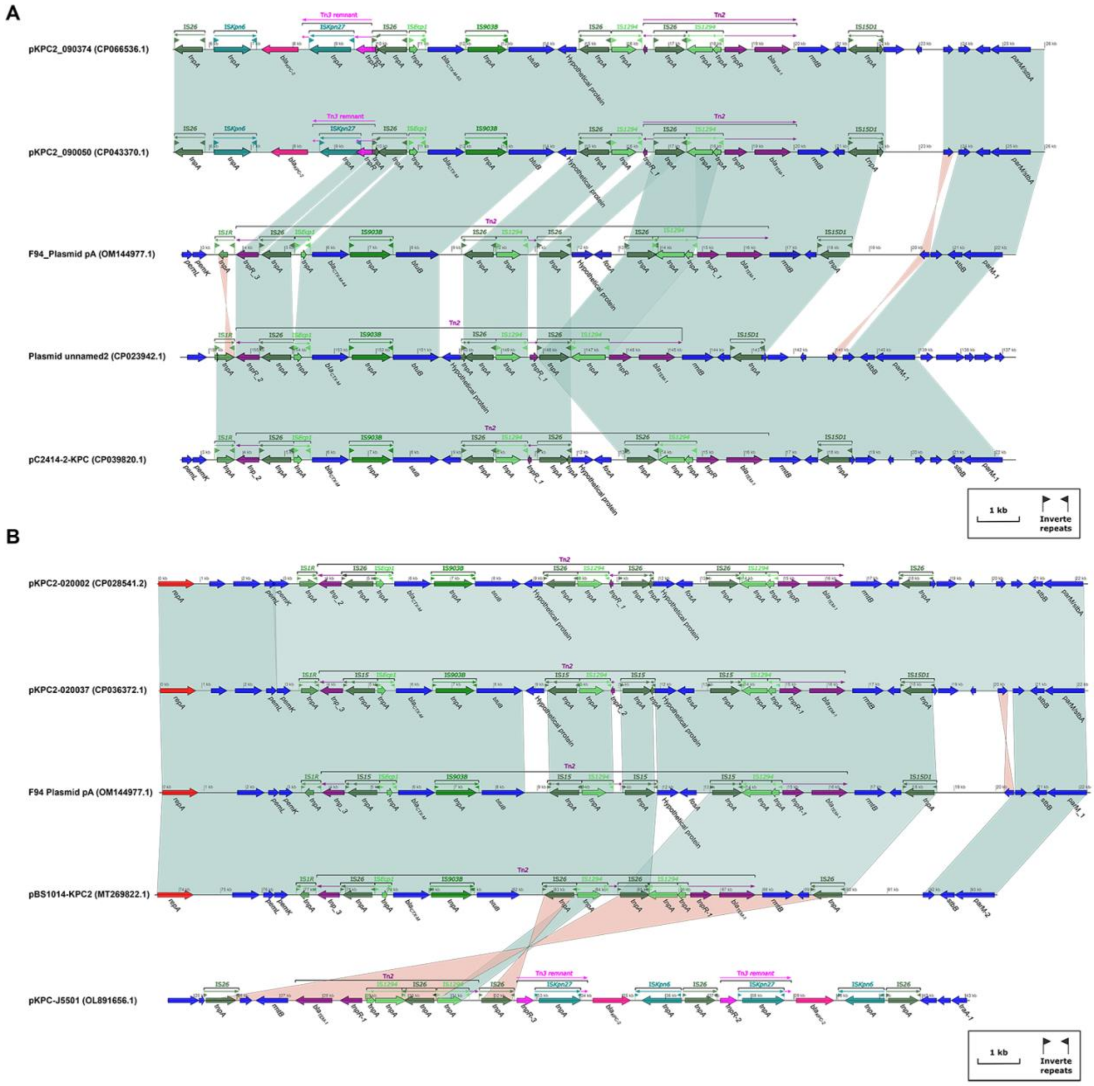

Fig. S2 Comparison of MDR (MDR1) regions in F94_plasmid pA with those of other plasmids. A, Comparison of MDR1 regions in F94_plasmid pA with those of related plasmids, such as pKPC2_090050, pKPC2_090374, Plasmid unnamed2, and pC2414-2-KPC; B, Comparison of MDR1 regions in F94_plasmid pA with those of related plasmids, such as pKPC2-020002, pKPC2-020037, pBS1014-KPC2, and pKPC-J550. This diagram was created by the R pacakge genoPlotR v0.8.11 software (http://genoplotr.r-forge.r-project.org/).

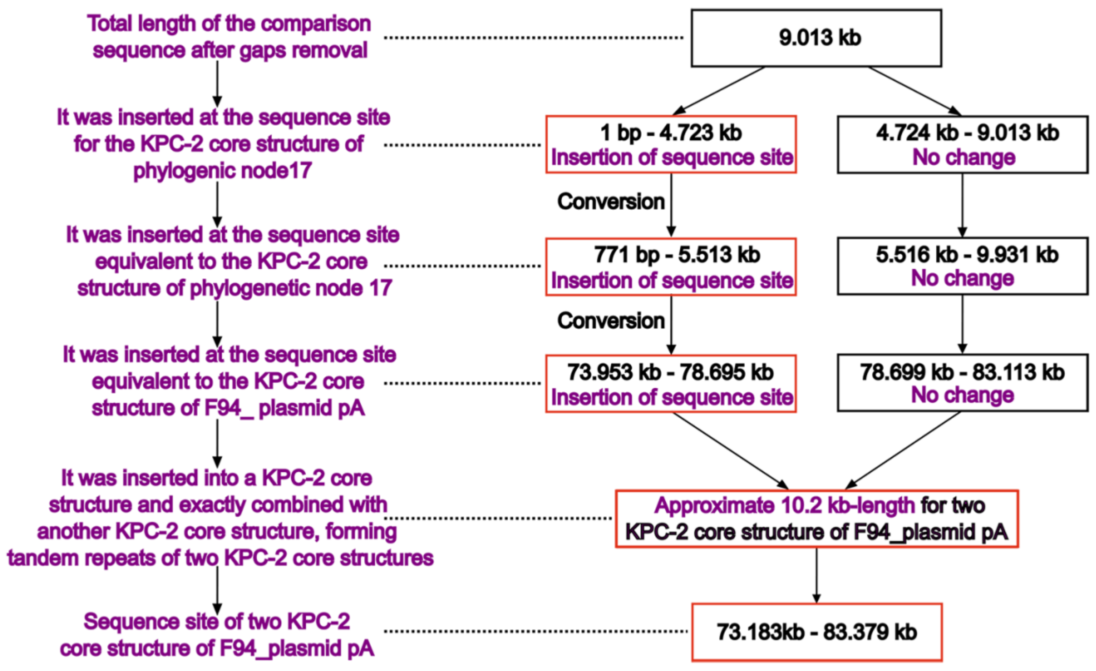

Fig. S3 Analysis and interpretation of gene sequence recombination events.
